# Care Deliberations of Family Carers of People Living With Dementia—Applying an Affective‐Discursive Practices Approach

**DOI:** 10.1111/1467-9566.70194

**Published:** 2026-04-30

**Authors:** Marja Lönnroth, Ulla Halonen, Emilia Leinonen, Lina Van Aerschot

**Affiliations:** ^1^ Faculty of Social Sciences University of Helsinki Helsinki Finland; ^2^ Department of Social Sciences and Philosophy University of Jyväskylä Jyväskylä Finland; ^3^ Faculty of Social Sciences Tampere University Tampere Finland

## Abstract

Dementia is the leading cause of care needs for older adults in Finland, with significant care contributions from families. Although not legally obliged, families often provide care driven by emotional bonds, moral obligations and practical considerations. Even though previous research has extensively explored the reasons why families provide care, analyses of their affective dimensions remain scarce. This study aims to explore these by examining the care deliberations of family carers of people living with dementia, using an affective–discursive practices approach. It focuses on how the entangled dimensions of affect, embodied experience and discourse shape their care choices, applying the approach to the written diaries of 15 Finnish adult children of people living with dementia. The approach embeds personal experiences and interactions within broader sociocultural structures. The results show how carers draw from societal norms and expectations linked to family care (e.g., devotion, duty and reciprocity) but also how they might renegotiate them to justify withdrawing from care. The analysis highlights how affective dilemmas can be approached by linking affect to discourse on a macro level, looking at societal discourses and the affective climates of care and examining how affect is mobilised in everyday interactions to make sense of care choices.

## 1 Introduction

Dementia is the main reason that older adults need care in Finland (Leppäaho et al. 2019). In addition to professional care services, people living with dementia receive a significant amount of help from their families. Previous research has shown that such family care is demanding, as it requires presence and responsibility for dealing with tasks that the person living with dementia can no longer manage independently (Halonen 2023). In Finland, all individuals in need of assistance are entitled to care services, which are provided based on a needs assessment. However, due to services being restructured to bring them into line with the ideals of deinstitutionalisation, refamilialisation and marketisation of care—the aim of which is to reduce public responsibility for care provision—the number of places with round‐the‐clock care has been reduced, and access to home care has been limited (Kröger 2019). This has led to a greater number of older people with severe conditions living at home and to the responsibility for providing the care that is no longer provided by formal services, being transferred to family members (e.g., Vaiva‐kollektiivi et al. 2020; Halonen 2023).

A population survey conducted in 2022 showed that 1.2 million—a quarter of all Finns aged over 20—periodically helped or cared for a relative or friend with an illness or weakening functional ability to enable them to continue living independently at home (Ilmarinen et al. 2023). Dementia can disrupt many everyday activities that we take for granted. Cognitive decline and loss of short‐term memory bring about a lack of control and predictability, not only for the person living with dementia but also for their families (Cipriani et al. 2020). In this respect, dementia is a social illness, as it profoundly impacts the lives of family members as they see the person living with dementia gradually lose their cognitive capacities or witness changes in their personalities. Dementia can alter the relationship dynamics between spouses and between generations (Van Aerschot et al. 2025), which may, in turn, affect the carer's other relationships, working life and well‐being. These impacts and challenges prompt an important question: Why do family members assume caregiving responsibilities despite the potential difficulties involved—especially considering that, in many countries, including Finland, they are under no legal obligation to do so?

Previous studies have explored this question extensively and found that the motives for providing care are multiple and complex (for reviews on informal care, see Zarzycki et al. 2023; on care for persons living with dementia, see Greenwood and Smith 2019; on Finland, see Leinonen 2011). They range from emotional and familial bonds to moral obligations, expectations and practical considerations, such as the costs of formal care and perceptions of its poor quality (Greenwood and Smith 2019; Zarzycki et al. 2023).

What these and other studies of care motives show is that our choices are often deeply emotional and reflect the general meanings attached to care, illness and mortality (Cromby 2011). Despite this clear emotional dimension, studies of the affective aspects of care deliberations remain scarce. Moreover, because negotiations about care choices are complex and involve intricate discursive work and various dilemmas and contradictions (L. Sointu 2018), we also need to consider the discursive practices through which we make sense of care choices. Guided by this premise, this article examines how family members of people living with dementia make sense of their care choices. We analyse diaries written by 15 adult children to answer the question: How do family caregivers make sense of their care choices and justify them, and how is affect mobilised in their sense‐makings?

In this article, we focus particularly on the affective–discursive practices through which care deliberations are negotiated. We have chosen this perspective because an examination of only the discursive may overlook the full emotional resonance of illness and care (E. Sointu 2016). Moreover, Research has traditionally focused on the dyadic relationships between the carer and the person needing care (Purkis and Ceci 2015), exploring how well‐being is affected at the individual and interpersonal levels. Our approach aims to broaden the perspective of research on family care, zooming out from the immediate care relationship to also consider the broader sociocultural structures and normative expectations that frame this relationship (Innes 2009; Tolhurst et al. 2017).

To expand on this, we draw on insights from feminist care literature (Tronto 1993; Twigg 2000; Bowlby et al. 2010; Weicht 2015) focusing on the situated, relational and emotional dimensions of care practices and embedding these experiences and interactions in the care relationships within sociocultural structures. Integrating these insights into the affective–discursive practices framework developed by Wetherell (2012; Wetherell et al. 2015) enables us to simultaneously explore care as an affective experience and as a discursive practice. This article thus aims to create an understanding of family carers' care choices that goes beyond the immediate caring relationship by considering the discursive regimes and affective climates in which these relationships are embedded. We argue that these affective dimensions, embedded in broader societal understandings of care, have a significant impact on how carers make sense of their own often emotionally ambivalent situations. Paying attention to the affective dimensions of care in their deliberations thus gives us a clearer idea of how care comes to matter in their lives (Venäläinen 2022).

## 2 Care as Affective Practice

Traditionally, care has been approached from two distinct but complementary angles: care as a material entity, that is, the physical labour it involves; and care as an emotional entity, encompassing its affective aspects (see, e.g., Graham 1991; Twigg 2000). This distinction, particularly the emotional aspects of caring for someone, has marked it as a private activity (Graham 1991), almost exclusively located within the private sphere of the home. Yet, carers are linked to the institutions, policies, legislations and discourses that surround dementia and care (e.g., Heaton 1999; Øydgard 2017; Purkis and Ceci 2015). Care is thus not only a private matter, nor are the emotions and affects that shape our care choices (Ahmed 2014a; see also Sihto 2018). The positive emotional bond that is expected to exist between family members, for instance, can impose a sense of duty and responsibility (Greenwood and Smith 2019). Family care has even been described as a labour of love that is expected to continue after the love has ended (Ulmanen 2015). Therefore, not being able to live up to these expectations might result in feelings of guilt and discomfort (Bowlby et al. 2010).

Benner and Gordon (1996, 43–44) conceptualise care practice as a ‘culturally constituted, socially embedded way of being in a situation and with others’. This practice consists of embodied actions, emotions and sentiments and of responses to these, which together form an endeavour to contribute to the well‐being of another person (L. Sointu 2018). These embodied sensations, despite being sensed in the individual body, are nevertheless based on a shared cultural understanding of what is an appropriate response or action in the specific context (L. Sointu 2018), that is, what kinds of affects and emotional reactions the surrounding discursive climate enables (Venäläinen and Calder‐Dawe 2024). To study exactly how these embodied affects, feelings as emotion labels and discourses of caring interrelate (L. Sointu 2018), we approach the care practices via the affective–discursive practices framework developed by Wetherell (2012).

Affective–discursive practice refers to the repetition of social action in which the affective and embodied dimensions of being and doing enmesh with the prevailing discursive structures (Canada et al. 2021). Wetherell's approach considers this interweaving of affect and discourse a form of embodied meaning‐making; that is, the bodily sensation, the affect, is combined with the subjective experiences of feelings and communicated through emotion labels that can be articulated and understood as sociocultural constructs (Wetherell 2012, 19–20; Venäläinen and Calder‐Dawe 2024). Thus, affect should not be treated separately from discourses surrounding dementia and care, as it often constitutes the very core of the experience of living with this condition.

L. Sointu (2018) applied an affective practice approach in her study of spousal dementia care. She highlighted the tensions that arise from shifting from an intimate relationship to a care relationship and showed that attending to the care needs of a person living with dementia requires exercising power in ways that may differ from both the ideals and reality of a spousal relationship. She shows how the spouses' expectations of intimate partner relationships lead them to manoeuvre through conflicting discourses and emotional ambivalence. Her conclusions indicate that in addition to benevolent acts of affection, care may also entail conflict, contradictions and frustration (see also Tronto 1993).

In sum, the affective–discursive approach guides us towards an analysis that considers the emotive aspects of people's lives and the ways in which these are shaped by both historically continuous macro‐level discursive regimes and context‐specific micro‐level interactional patterns. The approach is thus particularly useful for shedding light on the links between wider societal tensions and the affective–discursive ambivalence of individuals (Venäläinen and Calder‐Dawe 2024). Accordingly, it enables us to acknowledge the broader relational aspects of care, instead of the narrower dyadic perspective of only the carer and the person living with dementia. It also enables us to look at caring as an activity that entails personal thoughts, feelings and involvement without assuming that these are always positive. It challenges the essentialist assumption that feelings or sentiments are somehow inherent to the individual, rather than jointly constructed by those who engage with them (L. Sointu 2018). Furthermore, focusing on the discursive and affective aspects of care choices allows us to envision the ways in which care practices come to matter in people's lives (Venäläinen 2022).

## 3 Data and Methods

### 3.1 Collection and Description of Data

The data for this study were gathered as part of a larger research project called “Living with dementia: Social relational perspective to sustainable care”, which aims to investigate what it is like to live with dementia and what kind of support people living with dementia and their carers need. The University of Jyväskylä's ethics committee approved the study (ethical approval number 498/13.00.04.00/2023, issued on 24 April 2023).

We searched for participants aged under 68 who considered themselves to be carers of a home‐dwelling person with dementia but who did not necessarily have an official family care agreement. Information on the study and a call for participants were published in a media bulletin posted on the project's website and on social media (Facebook, Instagram, X and LinkedIn). They were also distributed to various NGOs related to family care. We did not limit the sample size and included everyone who expressed an interest in participating.

Participants were emailed information about how their personal data would be used in terms of data protection, their rights and the purpose of the study. The invitations to write diaries asked potential participants to write about certain core features of their thoughts, emotions and experiences, allowing them to express their reflections freely (Bartlett and Milligan 2021)—for instance, how caring for a person living with dementia had affected their social relationships or working life. A short guide for writing the diaries used cues such as ‘What is it like to be the family member of someone living with dementia?’, ‘What choices do you have to make in your life because someone in your family has dementia?’ and ‘How do you combine your own life with supporting your family member with dementia?’ The diary writers were also asked to reflect on their past experiences, their present daily life and the future and to provide background details on their age, work and education, their relationship with the person living with dementia and their geographical distance from them. The participants were free to choose how much and how often they wrote. They were told that they could return the text whenever they felt it was ready, but at the latest 3 months after receiving the instructions. The researchers had no contact with the participants during the diary‐writing period; thus, the only prompts they received were those mentioned above.

By the end of December 2023, we had received 21 computer‐written diaries: 4 either printed or sent by regular post, and 17 in a digital format sent by encrypted email. Some texts had been composed in a single sitting and thus were more like a response to a writing prompt, whereas others had been crafted gradually in a diary‐like fashion over an extended period. The length of the texts varied from 2 to 25 pages. Also, the style of writing varied—some of the participants had chosen to focus on everyday practical things in their lives, whereas others had written lengthy entries on their current moods and reflected on their relationship with the person living with dementia and their care and illness. Thus, the amount of affective potentialities displayed in the replies varied. This might be because some were more comfortable with writing and generally had experience of writing diaries. Moreover, the carers' situations varied in that some were actively caring for the person living with dementia at the time of the study, whereas for others, the person living with dementia was already deceased and the writers were reflecting on their relationship history. We expected different styles of writing, as we acknowledged that people communicate their experiences differently and choose to write about what they consider to be important at that moment (Spowart and Nairn 2014).

**FIGURE 1:**
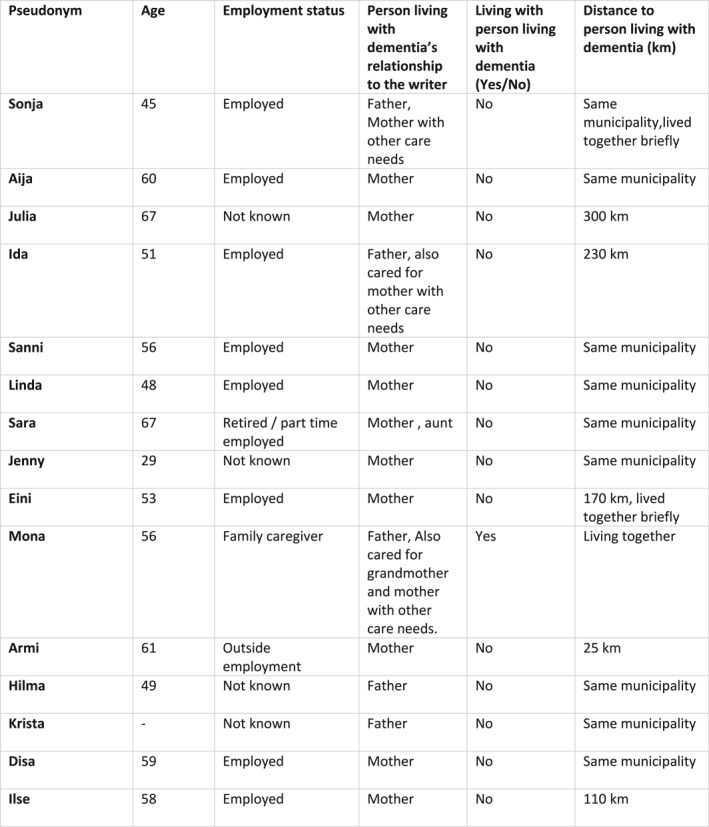
Information about the participants.

The writers were either the adult daughters (*N* = 15) or the spouses (*N* = 6) of the person living with dementia, but some had also taken care of other relatives such as grandparents or aunts. For the purposes of this article, we decided to include only the diaries of the daughters (for demographic information, see Figure 1). All the writers were thus women, which is not something we deliberately sought but which might reflect the gendered distribution of care responsibilities. It might also be because women are more inclined to express their thoughts in written form. Some had chosen not to provide care for the person living with dementia but still wanted to share their story of living with dementia and the emotional labour that it required.

The participants ranged in age from 45 to 67, which means that most of them were still working and some had children living at home. This is partly the reason we chose to include only daughters, as their situation differed from those of the spouses, who were mostly retired and whose children lived independently. This choice was motivated by the notion that the dynamics of their relationship with the person living with dementia might differ from those of spousal carers—the daughters had been cared for (to varying extents) by the person living with dementia while growing up, and now the relationship roles were reversed. Some daughters had had a strained relationship with their parents while growing up, and this relationship informed their sense‐making in the present. We wanted to capture this ambivalence. We also felt that the emotive aspects of adult children as carers have generally received less attention, as the focus of research has mostly been on the changing relationship between spouses (Halonen 2023).

Collecting data via diaries has certain benefits. Firstly, it captures a longer period of time than an interview, which is usually a single event. This offers the researcher insights into the fluctuating nature of emotions: their relationality, and how they arise, flow and shift between situations and context (Spowart and Nairn 2014). It enables some participants to document their thoughts and experiences closer to the time of the event, whereas for others, it offers a space in which they can reflect on their history and relationship with the already deceased parent or relative at their own pace. The diary method also gives participants more agency in the process—they write about what *they* deem most important, in contrast to other data collection methods. We also feel that it gives the participants more time to reflect on their thoughts and to choose the best time for sharing them—it might also be easier to share more sensitive experiences through writing, including the emotional ambivalences and dilemmas that we aimed to capture (Bartlett and Milligan 2021; Spowart and Nairn 2014).

It is worth noting that the participants did not write these diaries only for themselves; they knew that they were going to share their entries with the researcher and, through the researcher, possibly with a larger public. Writing enabled them to share their realities and experiences, to communicate their feelings, to try to make a difference and to tell us why, or why not, they care. Writing a diary for research purposes is thus a special kind of interactive context—it can give us insight into the ways in which family relationships and care issues are made sense of and the kinds of affective meaning‐makings that are possible and appropriate for the writer to convey in this situation.

### 3.2 Analysis

For the analysis, we applied Wetherell’s (2012) affective–discursive practices framework. Our reading of the data focused on three dimensions. First, we wanted to distinguish the ways in which the participants made care choices meaningful and justified them, that is, how they engaged with care as an emotional and material practice and how they justified this choice. Second, we wanted to identify the affective accounts of these choices, their functions and effects, and thus we focused on the affective capacities in the diary texts. We wanted to distinguish how the choices were informed by affects such as love or fear or by their past relationship history or their vision of the future and how this made them move either towards or withdraw from the relationship. Third, we examined how the participants' accounts related to the societal images of and discourses on dementia and care and how these had shaped their reasons, motives, thoughts, experiences and feelings (Wetherell 2012, 72–73; Venäläinen and Calder‐Dawe 2024). In other words, we were interested in the societal norms and expectations that shaped their understandings of care and the ‘appropriate’ emotional responses in relation to these expectations and how non‐normative affective practices, such as withdrawal, were legitimised. We thus aimed to shed light on how the participants aligned and misaligned themselves with normative expectations and conceptions of familial relations and care within families. Like Wetherell (2012; Wetherell et al. 2015), we see these practices as not merely symbolic but as also having material consequences because affect has the potential to incite action (Ahmed 2014a).

We began our analysis by carefully reading the material (15 diaries by the daughters) and then coded the data using the ATLAS.ti software programme, focusing on affect, emotion labels and the care‐related choices. In practice, this meant first coding the affective accounts, emotion labels and care choices that we detected in the diaries and then extracting them from the larger dataset. The next step was identifying the recurring ways in which the writers made sense of their care choices and how they mobilised affect in these sense‐makings (Venäläinen and Calder‐Dawe 2024). In other words, we wanted to explore how the writers legitimised and contested their choices and how they expressed their emotions and managed the affective dilemmas related to these choices. We then moved on to consider the broader social and cultural contexts that shaped the writers' care choices and how these were negotiated. Lastly, we explored the entwinement of affective and discursive practices to understand how social structures and relations both shape and are shaped by affect.

## 4 Findings

The analysis is divided into two sections according to how carers orient towards care responsibilities. In the first, we look at the choice to provide care, and in the second, withdrawal from care. In both sections, we show how these choices are managed and justified, using extracts from the data to illustrate our points. The extracts we chose shed light on the often‐dilemmatic nature of affective positionings and on the ways in which they are made sense of. We have given the participants pseudonyms to protect their anonymity. The extracts are translated from Finnish into English.

## 5 Choosing to Care: Expectations, Duty and Devotion

Previous research on the rationales of care has found reciprocity to be a norm or expectation (Zarzycki et al. 2023; Broom et al. 2016). Reciprocity was also a recurring theme in the diaries, as the participants wrote about returning the care they had once received. The accounts in which the writer complied with the expectations of care, both as an emotion and a practice, showed less of a need to do discursive work to justify the choice made, as exemplified by the extracts below:

In spite of everything, I think I’ve done my best and, most importantly for me, I’ve been able to return the care I received from those I love who gave me my roots and wings. It was a pleasure to accompany them to the end of their journey.

(Sonja, cared for her father living with dementia and mother with other care needs)

In her account, Sonja negotiates a sense of closure after both of the parents she cared for had passed away. Relying on the popular imaginary of informal care as a labour of love (Peterie and Broom 2024), she describes how a sense of gratitude and love had guided her care choices, which she mobilises to construct care as a pleasant experience (see also Jolanki 2016; Sihto 2018). In this respect, she is disputing the discourse that caring for a person with dementia is a burden—even though the phrase ‘in spite of everything’ hints that this has not always been easy. Care is thus constructed both as an act of love and a way of reciprocating the care and guidance she has received—expressed through metaphors of receiving ‘roots’ and ‘wings’ from her parents. Reciprocity as an expectation, and particularly a feminine expectation, is also expressed in the extract below:

Our parents took care of us [in our childhood] and in my case they cared for my children too, when they were small; so it’s only fair that I help my mother now (my father passed away earlier) […] I’m somewhat bitter that my sister doesn’t see it in quite the same way.

(Disa, cares for her mother)

Disa justifies her care choices in terms of gratitude for the care she has received from her mother in the past and a sense of duty to care for her in return. Whereas Sonja framed the care she gave her parents in the past as a personally and emotionally important choice, Disa considers it more of a responsibility in the present. This is also the lens through which she makes sense of her sister's choice to withdraw from care.

Disa conveys her feelings about the tasks and responsibilities of caring not being equally distributed and expresses a degree of bitterness on behalf of both herself and her mother (see also Leinonen 2011), who according to Disa now should be on the receiving side of care. Through this, she is reiterating normative expectations of filial obligations, emphasised by the statement ‘*it's only fair*’ to pay their mother back for the care and help they received in childhood and later when raising their own children. Care within families is seen as a continuum, extending from one generation to another, often understood as a feminine obligation. Disa's sister, who does not comply with these expectations, is portrayed as ungrateful and thus morally dubious (Leinonen 2011).

The above quotes show how negotiating the reciprocity of care is informed by both societal expectations and individual experiences, the history of relationships within a family and with the person who now needs care (Leinonen 2011; L. Sointu 2018; Zarzycki et al. 2023; Sihto 2018). This is also the case for Hilma:

My father was never really a very active parent—he always took a minor role when it came to raising us kids because of work and so on. My mother took on most of the responsibilities for raising us and most of the other things in the family too. Now with my father’s diagnosis, it’s made me think more about how he was as a parent and what I may have missed. I’ve realised that dwelling on negative experiences and bitterness won’t get me anywhere though. Somehow, I feel I should care for my father now, because he is helpless and needs care.

(Hilma, cares for her father)

In Hilma's account, the choice to care for a person not considered that emotionally close requires some management of affective dilemmas. Looking back at the relationship with her father, Hilma has realised that he was never very present in her childhood, so the question of reciprocating care becomes more problematic; is she expected to pay back for care she herself has not received? To manage this dilemma, she expresses a sense of obligation to care for the vulnerable and helpless, which justifies her decision to care for her father. Her account conveys the common understanding that dementia renders people helpless and the moral obligation to tend to those in need as an urge or call. It seems that these moral obligations, as well as the changing relationship dynamics caused by the illness, might have given Hilma new interpretative frames within which to make sense of her stance, enabling her to move away from negative experiences and bitterness and towards empathy—something that she also sees as protecting her own well‐being. Thus, perceiving the relationship as one of care might enable her to see her earlier relationship with her father differently and to justify why she now cares for a person with whom she has previously had distant relations (see also L. Sointu 2018).

The excerpt below further exemplifies the meanings given to relationship histories and past experiences, but from a slightly different perspective. Ida is orienting towards her own ageing, envisioning what kind of care she would like to receive. She herself does not care for or have contact with her parent because the relationship is strained:

I hope I’m loved when I’m older. My biggest dream would be to live with one of my children and play with their children. As I see it, the better mother I am to them now, and the more I love their partners and welcome them into the family, the better my chances are of this dream being fulfilled.

(Ida, father living with dementia)

Ida's account orients towards the future, in which she mobilises the trope of the ‘endeared elderly’, who is surrounded by her loved ones, with generations living under the same roof. For Ida, family care is thus linked to sentiments such as emotional connectedness, love and attachment (see also Zarzycki et al. 2023). Expressed in the often‐cited phrase ‘having passed away peacefully surrounded by family and friends’, family care is central to the cultural imaginary of ageing well and a good death—evoking a sense of closeness, reciprocity and closure, which is also indicative of social success (Broom et al. 2016). Ida's negotiations of reciprocity rely on the belief that a family that is close will also care for each other. This is something she as a mother is responsible for ensuring, reflecting the gendered expectations of mothers to maintain the ‘mood’ at home (Ahmed 2014b). Moreover, Ida's account resembles both Broom et al. (2016) and Zarzycki et al.’s (2023) notions that duty and reciprocity are temporally located; this caregiving reciprocity can be both retrospective (in terms of ‘paying back’ to one's parents) and future‐oriented (in terms of ‘investing’ in one's children).

Thus, a sense of obligation, duty and responsibility guides care choices. Some respondents also expressed the moral obligation to care in negative terms—a duty imposed by social norms and traditions from which the carer cannot walk away. This was often because the carer could see no other alternative and because they felt moral emotions such as guilt (Zarzycki et al. 2023; Broom et al. 2016; Lönnroth et al. 2026). Consequently, caring can produce situations that clash with the carer's wishes and ideals. As mentioned earlier, caring is often seen not simply as ‘a labour of love’ but also as an inevitable moral responsibility. Sometimes people also fear the social or emotional consequences of choosing not to care (Bowlby et al. 2010; Sihto 2018; Lönnroth et al. 2026):

Has anything good come out of this memory disorder? Well, right now I visit my mother more often, though I’m not sure it’s such a good thing. I might have a guilty conscience that I am trying to mend. I’m not very ‘proactive’ about visiting people. I’d prefer to get back to the comfort of home after work and do nice everyday things. Too much activity or being in a rush all the time just makes me feel restless.

(Aija, cares for her mother)

In this extract, Aija is explicitly responding to one of the writing prompts provided by the researchers: Has the memory disorder brought about anything good in your life? Elaborating on this, she is making sense of the changes in her life after making the choice to care for her mother. For Aija, the ‘good’ that has come from the illness is that she visits her mother more often now—something that is expected of adult children when their parents are ageing and with which she now complies. However, for her, this is not something unquestionably pleasurable. She finds herself in an affective dilemma, having to juggle the competing societal expectations of remaining independent and protecting her own well‐being with being a ‘good carer’ or ‘good person’ (Tolhurst et al. 2017). Although she sees the value in the time spent with her mother, she is not so sure about her underlying motivation for this and whether she genuinely wishes to be with her or if she is motivated by guilt. She positions herself as a person who enjoys solitude and admits that visiting her mother regularly goes against her personal preferences. This results in discomfort manifesting as a sense of restlessness, which she describes in her writing.

Some participants portrayed their motivation to care as stemming from practical considerations. They perceived that they had no option but to care, as care services were either nonexistent or insufficient (see also Lönnroth et al. 2026). Mona's excerpt illustrates this when she describes caring for both her parents despite an aversion to doing so. Both parents have had substance abuse problems and extensive care needs and sometimes react negatively and are hostile towards her.

I’d had it up to here [with my mother’s neediness] and so I alerted social services, telling them I can’t do it anymore. They said they would visit my mother in three weeks. THREE WEEKS? A woman who can’t walk to the post box, can’t go to the shop, can barely make it to a fridge that’s full of out‐of‐date food, who has cats that aren’t looked after, and whose bed and toilet are usually in a filthy state… So I swallowed my anger (yet again) and visited her a week later.

(Mona, cares for her father living with dementia and mother who has other care needs)

In her extract, Mona negotiates an affective dilemma, balancing between the societal expectations of the correct moral action and her strained relationship history with her parents. She elaborates on her past difficult relationship with her mother and the adversities she has encountered when caring for her, which forced her to cut off contact. Yet, as there are no other care options, she filters her emotional reactions and decides to ‘*swallow her anger*’. What she considers to be the right moral action weighs more in her decision‐making and justifies her choice to care for her mother despite the problems with which she has had to deal. She then channels her anger towards the services and the impossible situations into which the families are forced, listing the ways in which her mother is incapable of taking care of herself and underscoring this unreasonableness by using capital letters. By portraying her mother as someone who is unable to take care of basic things such as hygiene, she also mobilises a sense of disgust, which she needs to overcome, as she simultaneously sees her mother as someone in an extremely vulnerable position. Mona constructs her envisioned future as unpredictable, with possibly destructive consequences, and therefore has to act, despite the adversity she might be subjected to.

In sum, care deliberations and the affective dilemmas linked to them stem from relational considerations, not only in terms of the carer–cared‐for dyad but also in terms of the wider societal expectations regarding care and the material realities of how care services are organised. Societal images of dementia as an illness that causes extreme vulnerability and unpredictability, and the burden this places on families, further inform the negotiations of good care and moral action, as well as the appropriate emotional responses to them. These sense‐makings are informed by gendered expectations of care within families; it is often daughters who provide more hands‐on care and have to juggle multiple care responsibilities (Ulmanen 2015). They are also often expected to bear the brunt of emotional labour (Ahmed 2014b). These considerations also have a temporal aspect: The immediate care situation and the choices children make are informed not only by their relationship history with the person living with dementia but also by how the carers foresee the future of the person living with dementia, or their own future, with possibly growing care needs.

## 6 Withdrawing to Protect One's Own Well‐Being

The analysis revealed the ways in which the participants legitimised their choice to distance themselves from the relationship with the person living with dementia. This choice often required more justification and legitimisation, often by drawing on emotion labels with negative valence. Emotions such as fear or resentment, shaped by the past relationship or the personality changes caused by the illness, were prominent, as exemplified below:

The fear is so real, and I’m not exactly sure what it is I’m afraid of; it’s hard to ignore hatred and accusations from a person who played such an important part in my upbringing, even if I’m trying to do so in the diary […]. It’s hard to imagine that I even know this person whom I’m so fed up with, who I fear and try to protect myself from. Do I even have a father anymore, or has this terrible creature destroyed him?

When we last visited him, he attacked us. I really can’t answer his messages anymore, or call him or visit him either. Just the thought of it makes me feel ill, I get short of breath, my heart races and I start to shake.

(Ida, father living with dementia)

Ida’s account shows the complex interplay of affect and discourse and their material consequences. Ida does not meet the conventional expectations of care reciprocity, love and benevolence between adult children and their ageing parents. Instead, the physical aspects of fear and resentment, which she describes using embodied metaphors in her writing, have made her cut ties with her father. Her body stops her from physically approaching him, as she envisions a risk of both physical and psychological harm. This risk of injury is central in creating fear, which, in turn, incites action—moving away from the threat (Ahmed 2014a; Venäläinen and Calder‐Dawe 2024). For Ida, withdrawing from contact with her father is essential to protect her own well‐being, and this self‐protection serves as a justification for not adhering to the expectations linked to care.

However, Ida needs to negotiate an affective dilemma—she recognises her father's importance in her life but has to justify her lack of contact due to the changes in his behaviour and personality. Again, we can see how the meanings attached to care are linked to the personal past of the writer, their relationship with the person living with dementia and the new adversities that have come with the illness. Although the negative aspects of her present experiences with her father clearly tip the scales towards justifying her choice not to be in contact, she also acknowledges his importance in the past. She manages this dilemma using the metaphor of a creature taking over and destroying her father, making him a stranger to her—this is how she can legitimise her withdrawal.

Sara, who cared for her mother and two aunts living with dementia, also justifies the choice she made to withdraw from providing care for one of her aunts by describing the discomfort that the changes to her personality and her declining cognitive abilities had caused in the last few years of her life. She legitimises her choice by the need to protect her own well‐being:

She [aunt] lived to be almost 98, though in her final years she was so much in her own world that I couldn’t tell if she heard or understood anything. During COVID, I needed to stay 3 m away from her when visiting, and then she didn’t recognise me and couldn’t work out what I was saying. I’d basically already lost her a few years before she finally passed away, and I stopped visiting her because the visits just made me sad and I’m not sure if she got anything out of them.

(Sara, cared for her mother and aunt with dementia)

Sara's account of her aunt's cognitive abilities and not being able to communicate with her portrays the situation as her having lost her aunt already a few years before her actual death. This impaired communication and Sara's frustration at her aunt not responding to her efforts seemed to defeat the purpose of her visits and thus legitimised her withdrawal. In other words, Sara justified prioritising her own well‐being by drawing on the understanding of advanced dementia as a state of ‘living in one's own world’, where her efforts to communicate did not seem to benefit either of them (see also Kitwood and Bredin 1992, 285).

The legitimisation to withdraw thus comes from a desire to protect oneself from physical or psychological harm: Ida cites fear, whereas Sara wanted to avoid sadness from the loss of what she considered a meaningful connection. In the next example, Eini cites a lack of emotional resources:

I was very tired of the situation by that time, and in the spring of 2020 I didn’t visit my mother for several months. I guess I was also suffering from an illness myself that drained me emotionally—I felt unable to take it anymore. So, when the visits were forbidden for a while due to the COVID restrictions, I was actually relieved. Now I COULDN’T go there anymore! I was ALLOWED to stay at home! I had to stay at home!.

(Eini, cared for her mother)

Eini's account shows that she is still negotiating her choice to withdraw from providing care and the underlying feeling of guilt for not fitting in with society's expectations of care, even though her mother had already passed away. In remembering the care relationship, she goes back to the exhaustion she felt, which made her unable to visit her mother. Thus, she still feels obliged to justify her withdrawal in terms of being ill herself and the emotional resources required of her being drained. The COVID‐19 restrictions seemed to serve as a more ‘acceptable’ reason for withdrawal, giving her an external justification for not visiting her mother. She expressed this as a relief, which she highlighted by using capital letters and exclamation marks—now society was actually encouraging her *not* to visit.

Sonja similarly describes balancing her own well‐being and what she interprets as being the wishes of her father:

This [move to a care home] was an enormous disappointment for me. I felt I had also betrayed my father’s trust by not being able to organise care for him in the way he’d have preferred [at home] or would have been the best—and most humane—way for the last weeks of his life. At the same time, I knew I had reached my own limits. I also felt relief that there was no more I could do.

Even though I thought I knew my father and that I was on top of the situation, after just one night caring for him at home I knew it would require more than one person. What also weighed in was that I didn’t want my own children having to walk on eggshells at home because of grandpa or worrying about coming home after school when he’d had a particularly bad day. I also wanted them to have good memories of him, not to remember him as disorientated and unpredictable.

(Sonja)

Sonja, who, in an earlier extract, discussed the emotional importance of caring for her parents until their death, eventually had to make the decision to move her father to a care home, which she articulates as an enormous disappointment. Sonja's sense‐making of the transition to a formal care home tallies with the findings of Broom et al. (2016), who showed that a move to inpatient care is often articulated as an abandonment or failure. Sonja's expression of disappointment might also reflect the broader societal understanding of ‘ageing well’ and what constitutes ‘a good death’ (i.e., living at home until the end; see Broom et al. 2016). Zarzycki et al. (2023) also note that sometimes caring for the person living with dementia at home is motivated by a desire to protect that person's dignity and self‐esteem, something that care home arrangements are assumed to diminish. Sonja describes her sense of failure to do so as a betrayal, reflecting the moral dilemmas many carers face when trying to balance the wishes of the person living with dementia to stay at home and ensuring their safety and well‐being by moving them to a care home (Lönnroth et al. 2026).

Despite this sense of failure, Sonja justifies her choice to move her father into a care home by explaining that she reached her ‘*limits’* in terms of emotional resources. As well as her own well‐being, she cites that of her children as a motive for making her choice, reflecting the multiple caring responsibilities women often must juggle. She does not want their home to become a place of unpredictability and wants her children to cherish memories of their grandfather as he was before his illness—the person they knew before dementia changed him. By adjusting the source of tension, that is, the unpredictability caused by the illness, Sonja was protecting the mood at home—a gendered responsibility often expected of women (Ahmed 2014b). By withdrawing from care, she was able to keep the atmosphere at home safe and thus better maintain her own well‐being and that of her children.

In sum, daughters need to manage the affective dilemmas of not conforming to gendered societal expectations of care and familial relationships. The moral imperative to care for the vulnerable is particularly pronounced in the context of dementia, as the illness is associated with extreme dependency and loss of agency. Thus, carers often need to engage in affective–discursive work to negotiate and legitimise their choice of withdrawal by citing a sense of discomfort or feelings of fear or resentment. These negative effects can stem from both the relationship history and the present challenges of complex care needs, but they can also arise from envisioning the (unwanted) future with the progressing illness. The choice to withdraw from providing care is legitimised by the wish to protect one's own well‐being in the present as well as in this envisioned future. Often, the choice to withdraw is made sense of through moral feelings of guilt (Tolhurst et al. 2017; Sihto 2018), which still seem to need negotiating after the death of the person living with dementia and thus require affective work even in the present.

## 7 Conclusions and Discussion

This article has explored the affective–discursive practices of family carers of persons living with dementia as they make sense of their care choices. Our perspective leaned on insights from feminist care literature that highlight the importance of shifting the focus towards the relationality and situatedness of care practices and taking into account the significance of emotions and relationships in moral reasoning (Tronto 1993). We argue that the affective–discursive approach provided us with novel tools to try to understand these complexities.

Our analysis shows that these choices involve negotiations informed by gendered societal expectations and moral obligations, the temporal aspects of the relationship history with the person living with dementia and the envisioned future with the illness. Thus, the analysis unravels the complexities of not only care deliberations that may involve embodied and emotional discomfort but also those of love and connection. The affective complexities of care experiences, love, pain and attachment were entangled in unique ways in each of the participant's accounts. What we found interesting was the way in which the participants situated themselves within the broader cultural expectations that shape our understandings of dementia‐related care and how they were informed by their own life experiences and relational histories (see also Venäläinen and Calder‐Dawe 2024). The analysis thus highlights how carers can justify their care‐related choices and make sense of affects and their associated dilemmas or ambivalences by linking affect to discourse on both the macro level—drawing on societal discourses and affective climate of care and dementia, and the micro level—mobilising affect in interactions (Wetherell 2012; Venäläinen and Calder‐Dawe 2024).

We show how care practices are embedded in broader sociocultural expectations of reciprocal care and familial devotion or obligation, resembling what Peterie and Broom (2024) call the ‘moral economy of informal care’. These moral frameworks can suppress certain emotional expressions (e.g., reluctance to care, feelings of anger or estrangement), encourage others (e.g., empathy and reciprocity) and compel a person to care through mechanisms of blame and guilt. Thus, informal care can be seen as emerging from and reinforcing existing gendered social, political and economic hierarchies. This dynamic includes emotional tensions between connection and estrangement. Examining these tensions illuminates ‘how the scene of care creates trouble and tension rather than occupying a neutral moral territory’ (Peterie and Broom 2024, 60).

The analysis further shows how the writers negotiated gendered expectations, as women are often portrayed as ‘nurturers’ and expected to be responsible for maintaining close emotional relationships (Ulmanen 2015; Tolhurst et al. 2017). When they withdrew from the relationship, the daughters were compelled to justify their choices. This bears similarities to May's (2008) research on motherhood, which shows how mothers manage to uphold themselves as ‘good’ mothers, even when they have failed to fulfil the expectations of ‘proper’ mothering. As in May's (2008) study, it seems that, in addition to serving as a platform for sense‐making, these written accounts also functioned as a means of accounting for not adhering to societal expectations.

Indeed, the analysis highlights how affects (e.g., shame, guilt and resentment) are not only sensations (Wetherell 2012; Wetherell et al. 2015) but that they also reflect a person's perceived success or failure in adhering to societal norms and values (Sayer 2005). These emotions can thus be seen as the affective consequences of everyday norms related to different social roles, highlighting the intricate connection between affective and moral aspects in caring relationships (Broom et al. 2016). Consequently, the emotions expressed and described are understood not only as ‘inner states’ or emotion labels but also as reflections of how affect is embedded in social practices and how it contributes to the construction of social reality. Moreover, we underline that affective–discursive *practices* are indeed practices, with material consequences. For example, fear makes people withdraw from something or someone, but pity or empathy incite people to change a situation for the better (Ahmed 2014a; Venäläinen and Calder‐Dawe 2024). In this way, affective–discursive practices are concrete in that they make carers either engage or withdraw from care.

At this point, we should mention the ethical considerations related to the distress that writing diaries such as these may cause. As became evident in some of the above extracts, the act of writing caused some participants discomfort. However, for others, the diaries provided a positive outlet for reflecting on their situation and making sense of their thoughts and feelings. We admit that we could have better taken the possible distress into account during the process. For example, we could have provided information on where to find support if the anxiety became overwhelming. Yet, replying to the call for written, individual diaries was a choice that the participants were able to make several times during the process. We made it easy for them to decide to stop writing or not to send their written material.

Finally, we turn to the limitations of this study. Firstly, the data came from a limited number of participants, within a specific cultural and sociopolitical context, and focused on their lived experiences with the illness. Thus, the results cannot be generalised to other contexts. Moreover, the data were somewhat biased, as the participants formed a rather homogeneous group: only women belonging to the majority population who were mainly relatively highly educated and could write well in Finnish. We speculate that this might also reflect the gender disparity in family care arrangements (see, e.g., Vaiva‐kollektiivi et al. 2020; Ilmarinen et al. 2023). With this in mind, we recommend that future research explores a more heterogeneous group of caregivers and their intersectional privileges and disadvantages as family carers.

Despite these limitations, we argue that our analysis sheds new light on the affective–discursive resources that family carers draw upon when making sense of their lives with care responsibilities. It demonstrates an approach that seeks to consider the influences of the subjective, interactional and wider societal factors that shape our understandings of care (as suggested by Tolhurst et al. 2017). In line with Tolhurst et al. (2017), we argue that to profoundly understand why and how informal care requires social and emotional resources and often becomes burdensome, we have to ‘lift our gaze’ from the care dyad and consider the wider social contexts in which decisions on caring are made. Moreover, by highlighting the affective dimensions of care, this article has focused on the ambivalences of caring as a lived complex experience and how these are managed. With this, it offers a nuanced insight into the intricate relationship between the cultural understandings related to family care and the subjective experiences of those who are expected to provide care.

## Author Contributions

**Marja Lönnroth:** conceptualization, investigation, writing – original draft, methodology, writing – review and editing, formal analysis. **Ulla Halonen:** conceptualization, funding acquisition, writing – original draft, writing – review and editing. **Emilia Leinonen:** conceptualization, funding acquisition, writing – original draft, writing – review and editing. **Lina Van Aerschot:** conceptualization, funding acquisition, writing – original draft, writing – review and editing, project administration.

## Funding

Kone Foundation, Project: Living with dementia: Social relational perspective to sustainable care. Research Council of Finland, grant number: 354791.

## Ethics Statement

We applied for a preliminary ethical assessment to determine whether a full ethical review would be necessary for our research project. After reviewing our application, the University of Jyväskylä's ethics committee concluded that there were no ethical obstacles to proceeding with the study as planned (ethical approval number: 498/13.00.04.00/2023).

## Conflicts of Interest

The authors declare no conflicts of interest.

## Data Availability

The data that support the findings of this study are available upon request from the corresponding author. The data are not publicly available due to privacy or ethical restrictions.
